# Clinical risk factors of licorice-induced pseudohyperaldosteronism: a 2026-updated narrative review

**DOI:** 10.3389/fphar.2026.1840515

**Published:** 2026-05-28

**Authors:** Tetsuhiro Yoshino, Toshiaki Makino

**Affiliations:** 1 Center for Kampo Medicine, Keio University School of Medicine, Tokyo, Japan; 2 Department of Pharmacognosy, Graduate School of Pharmaceutical Sciences, Nagoya City University, Nagoya, Japan

**Keywords:** 11*β*-hydroxysteroid dehydrogenase type 2, 3-*epi*-18*β*-glycyrrhetinic acid, intestinal microbiota, kampo medicine, licorice, pseudohyperaldosteronism

## Abstract

Pseudohyperaldosteronism induced by the root of *Glycyrrhiza uralensis* Fisch. and *Glycyrrhiza glabra* L (licorice) is a frequent adverse effect of Japanese traditional Kampo medicines, characterized by hypokalemia and hypertension due to the inhibition of 11*β*-hydroxysteroid dehydrogenase type 2 (11*β*-HSD2). While daily licorice dosage is a primary risk factor, it often fails to explain inter-individual variability, where some patients develop pseudohyperaldosteronism at low doses while others tolerate high doses. This updated narrative review redefines clinical risk factors based on recent pharmacokinetic discoveries, specifically the identification of 3-*epi*-18*β*-glycyrrhetinic acid (3-*epi*-GA) and the role of intestinal microbiota. We reviewed recent pharmacological and pharmacokinetic evidence regarding the absorption, distribution, metabolism, and excretion of glycyrrhizin and its metabolites. We discuss the pharmaceutical factors altering the absorption of glycyrrhizin, one of the constituents of licorice, such as pH-dependent solubility in *Schisandra*-containing formulations and enzymatic competition by baicalin in *Scutellaria*-containing formulations. Importantly, we highlight the “epimerization phenotype,” where specific intestinal microbiota convert glycyrrhizin into 3-*epi*-GA. Unlike typical GA, 3-*epi*-GA is resistant to hepatic sulfation by sulfotransferase (SULT) 2A1, leading to prolonged accumulation. Furthermore, hypoalbuminemia increases free metabolite levels, potentially facilitating direct luminal access to renal tubules via glomerular filtration. Combined with the age-related decline in 11*β*-HSD2 activity and renal excretion via organic anion transporters, these factors create a high-risk metabolic profile. We propose a precision medicine approach for risk assessment that integrates formulation characteristics, host intestinal microbiota function, and physiological reserve, moving beyond simple dosage counting to prevent this iatrogenic condition.

## Graphical Abstract

**Figure:**
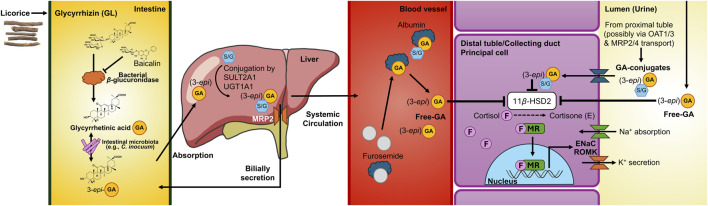

## 1 Introduction: traditional uses of Glycyrrhiza species, their bioactive constituents, and the risk of pseudohyperaldosteronism

Licorice (the root of *Glycyrrhiza* spp., Leguminosae) is one of the most ancient and globally consumed botanical resources. In East Asia, it serves as an essential crude drug in traditional medical systems, including Japanese Kampo and Traditional Chinese Medicine (Ding et al., 2022). In the Japanese Pharmacopoeia 18th Edition, its origin is registered as the dried root or stolon of *Glycyrrhiza uralensis* Fisch. or *Glycyrrhiza glabra* L., and the minimum content of its marker ingredient glycyrrhizic acid (GL) is not less than 2.0% (The Society of Japanese Pharmacopoeia, 2021). Japanese traditional Kampo medicine also uses it as the herbal component in the formulations. It is included in approximately 70%–74% of all ethical or OTC Kampo formulations approved by the Japanese Government. Beyond its role as a harmonizer among the contents of crude drugs, the primary bioactive constituent GL and glycyrrhetinic acid (GA), one of the metabolites of GL by intestinal microbiota, possess significant pharmacological properties (Bakr et al., 2022; Shinu et al., 2023). These include anti-inflammatory, anti-allergic, and hepatoprotective effects, making them essential for treating chronic hepatitis, acute upper respiratory infection, and muscle cramps. Furthermore, GL is widely consumed as a natural sweetener in foods, confectionery, and chewing tobacco in Western countries, and a traditional thirst-quenching beverage during Ramadan in Middle Eastern countries (Mukhopadhyay and Panja, 2008).

However, the clinical utility of licorice is frequently overshadowed by its characteristic adverse effect: pseudohyperaldosteronism (Conn et al., 1968; Shimada et al., 2019). This condition manifests as hypokalemia, hypertension, and edema due to the inhibition of 11*β*-hydroxysteroid dehydrogenase type 2 (11*β*-HSD2) by GA, which allows cortisol to inappropriately activate mineralocorticoid receptors (Ploeger et al., 2001). Historically, risk management strategies have relied almost exclusively on the daily dosage of licorice. Epidemiological data, such as those from the Japanese Adverse Drug Event Report (JADER) database, indicate a statistical correlation between total licorice intake and the incidence of pseudohyperaldosteronism, leading to warnings for formulations containing more than 2.5 g of licorice per day (Kato et al., 2016) or even 1.125 g per day (Nakao et al., 2026). Yet, in clinical practice, relying solely on the “grams per day” metric is insufficient to evaluate the risk for the specific patient sitting in the examination room. There is a profound discrepancy between the prescribed dose and the individual’s susceptibility. For instance, shakuyakukanzoto, a Kampo formulation which contains a high dose of licorice (6.0 g/day) (Kato et al., 2016), is a well-known high-risk drug in Japanese traditional Kampo medicine. Conversely, shoseiryuto formulation, despite containing a relatively high licorice dose (3.0 g/day), presents a lower risk profile than expected because of the lower extraction efficiency of GL due to the acidic pH condition caused by the fruit of *Schisandra sinensis* (SF)*,* one of the components of shoseiryuto (Nose et al., 2017). In contrast, yokukansan formulation contains only 1.5 g/day of licorice—a supposedly “safe” low dose—yet it is frequently associated with severe hypokalemia and rhabdomyolysis, particularly in elderly patients with low body weight or hypoalbuminemia (Shimada et al., 2017; Ishida et al., 2020).

This paradox suggests that the risk of pseudohyperaldosteronism is not defined merely by the dose of licorice ingested but is driven by a complex interplay of pharmaceutical factors (extraction efficiency, pH of the decoction) and the host factors (drug metabolism and excretion). Recent research has identified that the intestinal microbiota plays a decisive role, not only in hydrolyzing GL to absorbable GA but also in converting it into stereoisomers such as 3-*epi*-GA (Sakoda et al., 2024; Sakoda et al., 2025). The ability of a patient’s specific intestinal microbiota to produce these active metabolites, combined with their age-related decline in 11*β*-HSD2 activity and renal excretion capacity, creates a unique “metabolic phenotype”. Therefore, a modern risk assessment must move beyond simple dosage counting to a precision medicine approach that considers formulation chemistry, individual metabolic capacity, and physiological reserve. We previously reported on the clinical risk factors of licorice-induced pseudohyperaldosteronism from the perspective of such individual differences (Yoshino et al., 2021). This article serves as an updated narrative review, incorporating the significant research progress made over the past 5 years to elucidate the molecular mechanisms underlying these clinical risks. This updated narrative review incorporates literature published up to March 2026. We searched PubMed and Google Scholar using the primary keywords “licorice,” “glycyrrhizin,” “pseudohyperaldosteronism,” “intestinal microbiota,” and “pharmacokinetics.”

## 2 11*β*-hydroxysteroid dehydrogenase type 2 (11*β*-HSD2): the guardian of the mineralocorticoid receptor and its failure

**FIGURE 1:**
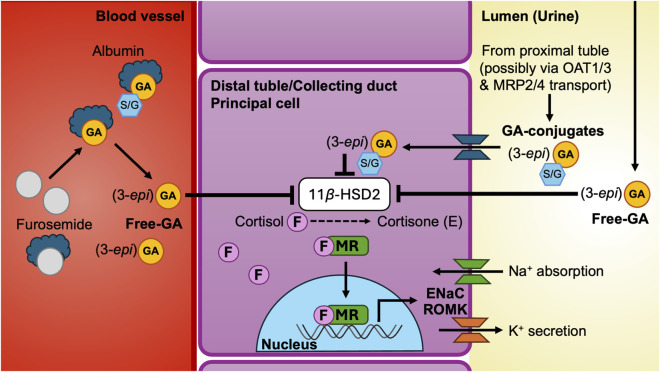
Mechanism of 11*β*-HSD2 inhibition and pseudoaldosteronism in renal distal tubule cells. In the blood, glycyrrhetinic acid (GA), its stereoisomer (3-*epi*-GA), and GA-conjugates (e.g., GA-3-sulfate) are primarily bound to albumin. Loop diuretics, such as furosemide, compete for albumin binding sites, displacing GA and increasing the concentration of free GA, which enters the principal cells from the basolateral side. Simultaneously, the free fraction of GA and 3-*epi*-GA filtered by the glomerulus reaches the tubular lumen and enters the cells via passive diffusion. Additionally, hydrophilic GA-conjugates (GA sulfate/glucuronide; GA S/G), which are secreted into the urine from the proximal tubules via OAT1/3 and MRP2/4, travel down the lumen and are reabsorbed into the distal tubular cells, possibly via OATP1A2 expressed on the apical membrane. Inside the cell, these metabolites inhibit 11*β*-hydroxysteroid dehydrogenase type 2 (11*β*-HSD2), preventing the conversion of active cortisol (F) to inactive cortisone (E). The accumulated cortisol binds to the mineralocorticoid receptor (MR), translocates to the nucleus, and upregulates the transcription of ENaC and ROMK, resulting in increased sodium reabsorption and potassium secretion. *Abbreviations*: GA, glycyrrhetinic acid; 11*β*-HSD2, 11*β*-hydroxysteroid dehydrogenase type 2; MR, mineralocorticoid receptor; F, cortisol; E, cortisone; ENaC, epithelial sodium channel; ROMK, renal outer medullary potassium channel; OATP, organic anion transporting polypeptide; OAT, organic anion transporter; MRP, multidrug resistance-associated protein.

The final mechanism of licorice-induced pseudohyperaldosteronism converges on the dysfunction of the renal enzyme 11*β*-HSD2. Previous evidence suggests that this dysfunction is driven not only by potent competitive inhibition by metabolites (including 3-*epi*-GA) (Takahashi et al., 2019; Ishiuchi et al., 2021; Sakoda et al., 2024; Sakoda et al., 2025) but also by the transcriptional downregulation of the enzyme (Tanahashi et al., 2002) and an age-related decline in physiological reserve (Campino et al., 2013). Understanding these multi-layered mechanisms is essential for grasping the core pathophysiology (Figure 1).

### 2.1 Physiological role: enzymatic protection

The mineralocorticoid receptor (MR) is responsible for regulating sodium reabsorption and potassium excretion (Terker and Ellison, 2015). Although its primary ligand is aldosterone, the MR has an equally high affinity for cortisol (a glucocorticoid; corticosterone in rats) (Hellal-Levy et al., 1999). Since the plasma concentration of cortisol is 100 to 1,000 times higher than that of aldosterone (Syed and Qureshi, 2012), cortisol would normally occupy and permanently activate the MR (Fuller et al., 2012). This would lead to a state of constant hypertension and hypokalemia, known as Apparent Mineralocorticoid Excess syndrome (Funder, 2017).

To prevent this, 11*β*-HSD2 acts as a gatekeeper (Chapman et al., 2013). This enzyme is specifically expressed in aldosterone-target tissues, such as the renal distal tubules and collecting ducts, colon, and salivary glands. 11*β*-HSD2 irreversibly converts active cortisol (which has a hydroxyl group at the C-11 position) into inactive cortisone (which has a ketone group at the C-11 position), which cannot bind to the MR. This mechanism protects the MR from the vast excess of cortisol, allowing it to respond selectively to trace amounts of aldosterone.

### 2.2 Potent competitive inhibition by metabolites

Pseudohyperaldosteronism is an acquired form of apparent mineralocorticoid excess caused by the inhibition of 11*β*-HSD2 by GL metabolites (Bendjilali-Sabiani et al., 2026). In *in vitro* studies using rat kidney microsomes, we and others have characterized the inhibitory potency of these metabolites (Monder et al., 1989; Makino et al., 2012; Morinaga et al., 2018; Ishiuchi et al., 2019; Ishiuchi et al., 2021; Sakoda et al., 2024). It has long been known that GA, the aglycone of GL, is a much more potent inhibitor than GL itself. Our previous data indicated that the half-maximal inhibitory concentration (IC_50_) of GA was 0.32 μM, while that of GL was 2.2 μM, confirming that hydrolysis is required for toxicity.

Recent updates have expanded the list of “culprits.” We found that 3-monoglucuronyl-GA (3MGA = GA3G) has an IC_50_ of 0.26 μM, showing similar potency to GA. Furthermore, our latest research identified that the major human metabolite 18*β* -glycyrrhetinic acid 3-*O*-sulfate (GA3S, IC_50_: 0.10 μM) and the stereoisomer 3-*epi*-GA (IC_50_: 0.17 μM) are equally or even more potent inhibitors than GA. This suggests that multiple metabolites contribute to the suppression of enzyme activity in the human body.

### 2.3 Suppression of enzyme expression (down-regulation)

The dysfunction of 11*β*-HSD2 is not limited to temporary competitive inhibition. Tanahashi et al. (2002) demonstrated in rats that chronic administration of GA (120 mg/kg/day for 2 weeks) significantly reduced both the mRNA and protein expression levels of 11*β*-HSD2 in the kidney (Tanahashi et al., 2002). This “downregulation” effect suggests that chronic exposure to licorice does not merely block the enzyme’s active site but decreases the number of enzyme molecules available. This finding offers a physiological explanation for why some patients require weeks or even months to recover from pseudohyperaldosteronism symptoms even after they stop taking licorice. The protective system is not just blocked; it is dismantled and requires time to be resynthesized.

### 2.4 Age-related vulnerability

Individual susceptibility to pseudohyperaldosteronism is strongly influenced by the baseline activity of 11*β*-HSD2. It is well established that the activity of this enzyme declines with age (Stewart et al., 1987; Campino et al., 2013). Elderly patients have a naturally lower “reserve” of 11*β*-HSD2 activity. Therefore, even low concentrations of licorice metabolites, which would be manageable for a younger individual, can easily exceed the inhibition threshold in the elderly, triggering MR activation by cortisol. This age-related decline in enzymatic defense is likely the primary reason why pseudohyperaldosteronism is predominantly observed in the older population.

### 2.5 Genetic polymorphism of 11*β*-HSD2

Harahap et al. found a mutation in the 11*β*-HSD2 gene in one patient raising suspicion of licorice-induced hypertension (Harahap et al., 2011). However, sequence analysis of the promoter and exon regions of the 11*β*-HSD2 gene in 953 Japanese individuals revealed that mutations were rare, with minor allele frequencies of less than 0.5%. Furthermore, no mutations causing apparent mineralocorticoid excess were identified in a Japanese population of hypertensive patients (Kamide et al., 2006). Miettinen et al. also found no significant association between 11*β*-HSD2 gene mutations and licorice-induced hypertension in a study of 30 patients, and that mutations in the epithelial sodium channel (ENaC) subunit gene, rather than 11*β*-HSD2, may increase the risk of licorice-induced hypertension (Miettinen et al., 2010). However, ENaC mutations are also rare, having been found in only two of 30 patients with PsA, suggesting that their role in the onset of PsA is limited.

## 3 Absorption: intestinal microbiota and formulation properties controlling bioavailability

**FIGURE 2:**
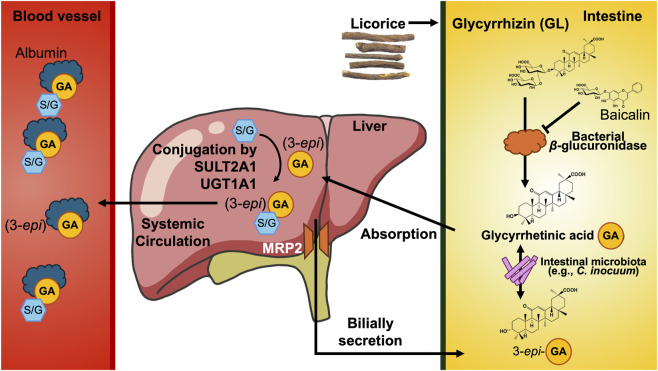
Pharmacokinetic pathway of licorice metabolites from intestinal absorption to hepatic disposition. Ingested glycyrrhizin (GL) is hydrolyzed to glycyrrhetinic acid (GA) by microbiotal *β*-glucuronidase in the intestinal lumen. This hydrolytic process is competitively inhibited by baicalin in formulations containing the root of *Scutellaria baicalensis* (SR). Specific intestinal microbiota, such as *Clostridium innocuum*, further convert GA into its stereoisomer, 3-*epi*-GA. After absorption into the portal vein, GA is metabolized in the liver by sulfotransferase 2A1 (SULT2A1) and UDP-glucuronosyltransferase 1A1 (UGT1A1) into hydrophilic conjugates (GA sulfate/glucuronide; GA S/G), which are excreted into the bile via multidrug resistance-associated protein 2 (MRP2). In contrast, 3-*epi*-GA shows resistance to these metabolic enzymes, leading to reduced biliary excretion and increased transfer into the systemic circulation. Abbreviations: GL, glycyrrhizin; GA, glycyrrhetinic acid; SULT2A1, sulfotransferase 2A1; UGT1A1, UDP-glucuronosyltransferase 1A1; MRP2, multidrug resistance-associated protein 2.

The absorption of GL, the primary bioactive constituent of licorice, is not a simple process of passive diffusion; it is a dynamic system regulated by the physicochemical properties of the formulation and the enzymatic activity of the host’s intestinal microbiota (Figure 2). Recent pharmacokinetic studies have clarified that the “prescribed dose” of licorice does not necessarily correlate with the “amount of active metabolic products absorbed.” In this section, we describe the pharmaceutical and host factors that drive this discrepancy.

### 3.1 Bacterial hydrolysis as the rate-limiting step

GL is a glycoside in which two molecules of glucuronic acid are bound to the C-3 position of GA. Because GL is hydrophilic and has a high molecular weight (822.93 g/mol) (The Society of Japanese Pharmacopoeia, 2021), it is poorly absorbed from the gastrointestinal tract in its native form. For GL to exert pharmacological effects or toxicity, it must reach the large intestine and undergo hydrolysis by bacterial *β*-glucuronidase (produced by species such as *Eubacterium* sp. GLH) to release the lipophilic aglycone, GA (Akao et al., 1987; 1988). Consequently, the hydrolytic capacity of the intestinal microbiota is the primary determinant of absorption. It is demonstrated that in germ-free rats or those treated with antibiotics, plasma GA concentrations drop to undetectable levels when GL was orally administered (Takeda et al., 1996; Ishida et al., 2022).

### 3.2 Pharmaceutical factors: why licorice content ≠ risk

Kampo formula is a mixture of multiple crude drugs, and the extract obtained by boiling them in water is usually used. Therefore, the amount of chemicals dissolved from the crude drugs into the decoction sometimes varies depending on the combination of crude drugs used. Therefore, there may not be a correlation between the amount of chemicals in the extract and the dose of crude drugs prescribed in the Kampo formula used. Furthermore, the interaction among crude drugs also occurs in the intestinal absorption phase. These findings revealed that the formulation matrix significantly affects GL exposure.

#### 3.2.1 pH-dependent solubility

Clinically, a Kampo formulation, shoseiryuto, contains a relatively high amount of licorice (3.0 g/day), yet it is associated with a lower frequency of pseudohyperaldosteronism compared to other formulations. Nose et al. attribute this to the pH of the decoction (Nose et al., 2017). Shoseiryuto contains SF, which is rich in organic acids (*e.g.*, citric acid, malic acid), lowering the decoction pH to below 4.0. Since the pKa of the carboxyl groups in GL is approximately 4.0–5.2, acidic conditions cause GL to shift from its ionized form to a non-ionized, molecular form, leading to precipitation and insolubilization. The concentration of dissolved GL in the shoseiryuto decoction is significantly lower than the theoretical value. This indicates that even if the daily dose of licorice is high, the actual amount of GL available for absorption is limited by the physical chemistry of the formulation.

#### 3.2.2 Enzymatic competition

Miyamura et al. (1996) reported that the hydrolysis of GL to GA was suppressed in Kampo formulations containing the dried root of *Scutellaria baicalensis* (SR), such as shosaikoto, compared to formulations without SR. Mechanistically, baicalin, one of the primary components of SR, acts as a potent inhibitor for bacterial *β*-glucuronidase. Kim et al. (1996) demonstrated that bacterial *β*-glucuronidase exhibits higher affinity for baicalin than for GL, leading to the preferential hydrolysis of baicalin. Building on this, a recent study by Nose et al. (2026) clarified that baicalin acts as a noncompetitive inhibitor of this enzyme, and they confirmed *in vivo* that removing SR from shosaikoto significantly increases serum GA concentrations in mice. Consequently, the presence of baicalin delays or limits the release of the active aglycone, GA. In contrast, formulations lacking SR, such as Kampo formulations of yokukansan (containing 1.5 g licorice/day) and shakuyakukanzoto, showed high hydrolysis rates. This explains why yokukansan can induce high blood GA levels and side effects despite its relatively low licorice content.

#### 3.2.3 Another source of plasma GA

Plasma GA is mainly derived from the metabolite of GL by intestinal microbiota. However, licorice contains another glycoside than GL that is metabolized by intestinal microbiota to produce GA. Miyoshi et al. reported that gala-GL, an isomer of GL having a galacturonic acid motif instead of a glucuronic acid motif at the terminal point of the sugar motif, was contained in Kampo extracts, and its content was about 7.3%–10.9% of GL (Miyoshi et al., 2026). The rate of hydrolysis of gala-GL to GA by intestinal microbiota was approximately six-times slower than that of GL, which might prolong GA absorption and contribute to the accumulation of total systemic GA.

### 3.3 Host factors: the “epimerization” phenotype

#### 3.3.1 Bacterial stereoisomerization (The 3-*epi*-GA pathway)

A critical update in our understanding is the discovery of the stereoisomerization of GA by intestinal microbiota. Specific microbiota, such as *Clostridium innocuum*, have been demonstrated that they do not merely hydrolyze GL but actively convert the resulting 18*β*-GA into its stereoisomer, 3-*epi*-GA via an oxidation-reduction pathway involving the intermediate 3-oxo-GA (Hattori et al., 1983; Hattori et al., 1985). We found that the ability to produce 3-*epi*-GA varies greatly among individuals (Sakoda et al., 2024). Importantly, we showed that patients with detectable serum levels of 3-*epi*-GA have a significantly higher risk of developing pseudohyperaldosteronism. This suggests that the presence of specific “epimerizing intestinal microbiota” is a key determinant of individual susceptibility.

#### 3.3.2 Transit time and sex differences

Factors that prolong intestinal transit time, such as constipation, increase the contact time between the drug and microbiota, thereby enhancing hydrolysis and absorption. Furthermore, bacterial enzymes involved in GL metabolism (3*β*-HSD, etc.) are originally responsible for the metabolism of endogenous steroids and bile acids (Ly et al., 2021). We hypothesize that the potential sex difference in pseudohyperaldosteronism incidence (higher in women) (Sabbadin et al., 2024) may be partly mediated by sex-hormone-dependent differences in intestinal bacterial metabolic activity, leading to altered production of GA and 3-*epi*-GA.

## 4 Distribution: albumin binding and the dynamics of free metabolites

The process of distribution is not a static process; it is a key factor in explaining individual susceptibility to licorice-induced pseudohyperaldosteronism. While previous studies focused primarily on the total blood concentration of GA, recent findings highlight that when the buffering capacity of albumin is compromised by aging, malnutrition, or competitive drugs (e.g., loop diuretics), free metabolites bypass standard distribution restrictions. This potentially facilitates filtration-dependent luminal entry as a potential delivery route to the target enzyme.

In serum, absorbed GA and its stereoisomer (3-*epi*-GA) exist primarily bound to serum albumin. GA binds mainly to Sudlow site I (or its vicinity) on the albumin molecule (Zhang et al., 2025). Under normal conditions, more than 99.9% of GA and 3-*epi*-GA exist as albumin-binding form in serum, keeping the concentration of free-form extremely low (Ishida et al., 1988; Sakoda et al., 2025). Since binding-form GA in serum hardly passes through the renal glomerulus and is not filtered into the urine, the concentration of GA in urine was very low and below the detectable level (Makino et al., 2012; Takahashi et al., 2019). Therefore, GA in the circulation is difficult to penetrate the cell membrane by passive diffusion, and can only be transported into cells by an active transporting system via the transporters that specifically transport GA; unless GA exists as a free-form which can be transferred into the cells by passive diffusion.

Furthermore, it is estimated that free-form GA passes through the glomerulus directly into the tubular lumen (apical side). We propose this as a filtration-dependent luminal entry mechanism. As the urine concentrates along the nephron, the concentration of free GA and 3-*epi*-GA in the lumen rises significantly. Since organic anion transporting polypeptides (*e.g.*, OATP1A2) are expressed on the apical membrane of the distal tubular cells (Roth et al., 2012), and since there is less albumin in primary urine, conjugated metabolite of GA may be actively reabsorbed via these transporters or free-form GA and 3-*epi*-GA may enter the cells by passive diffusion (Lan et al., 2021).

These processes would expose the intracellular 11*β*-HSD2 enzyme to high concentrations of inhibitors from both passive diffusion of aglycones from both basolateral and apical sides and the active uptake of its conjugates from the luminal side into renal epithelial cells.

### 4.1 Albumin binding saturation and competitive inhibition (drug factors)

The equilibrium between free- and binding-form of GA in serum albumin is easily disrupted by concomitant medications. Loop diuretics (*e.g.*, furosemide) are of particular concern. Zhou et al. (2008) reported that furosemide has a high affinity for albumin site I and competes with GA for binding (Zhou et al., 2008). In the presence of high concentrations of furosemide, GA is displaced from albumin, causing a significant increase in the free GA concentration. Notably, thiazide diuretics (*e.g.*, hydrochlorothiazide) do not cause this displacement. This suggests that the established clinical risk of combining licorice with diuretics involves not only the promotion of potassium excretion but also the drug-drug interaction that increases serum concentration of free-form GA through albumin displacement.

### 4.2 Hypoalbuminemia and the “filtration-dependent luminal entry” route (host factors)

Hypoalbuminemia, often associated with aging, malnutrition, or liver dysfunction, critically affects the distribution volume and tissue transfer of GA. A decrease in serum albumin concentration augments the level of free GA in serum and its distribution in epithelial cells (Sakoda et al., 2025), even if the total serum GA concentration in serum remains unchanged. Shimada et al. (2017) identified hypoalbuminemia as an independent risk factor for pseudohyperaldosteronism in elderly patients taking yokukansan (a formulation containing licorice 1.5 g/day). Hypoalbuminemia is also one of the risk factors of pseudohyperaldosteronism by the filtration-dependent basolateral entry mechanisms as described above.

### 4.3 Distribution characteristics of the stereoisomer (3-*epi*-GA)

The distribution behavior of the recently identified metabolite 3-*epi*-GA requires further investigation, but its structural difference likely influences its affinity for albumin and tissue distribution. Since 3-*epi*-GA is resistant to hepatic sulfation by sulfotransferase 2A1 (SULT2A1), it exhibits a prolonged half-life in the blood (Sakoda et al., 2025). This resistance likely leads to sustained occupancy of albumin binding sites and, over time, an increased potential for the free form drugs to accumulate in tissues.

## 5 Metabolism: hepatic detoxification and stereoselective resistance

Once absorbed into the portal circulation, GA and its stereoisomers reach the liver, where they undergo phase II metabolic reactions, being conjugated with glucuronic acid and sulfuric acid. This hepatic clearance step determines the total amount of active compounds that enter the systemic circulation. Recently, we have identified that this process exhibits significant stereoselectivity, where 3-*epi*-GA evades detoxification, leading to its accumulation in high-risk patients.

### 5.1 Re-evaluation of major metabolites: from 3MGA to GA3S

Historically, 3MGA (=GA3G) had been proposed as the major conjugated metabolite of GA and the causative agent of pseudohyperaldosteronism (Kato et al., 1995; Makino et al., 2008; Makino et al., 2012). However, using high-sensitivity LC-MS/MS analysis in human clinical studies, we found that the serum concentration of GA3G was extremely low or undetectable in patients with normal liver function (Takahashi et al., 2019). Instead, we identified GA3S, along with unmetabolized GA, as the dominant circulating metabolites in human blood. We also found that the major conjugated metabolite of GA was not GA3G but GA-30-*O*-glucuronide (GA30G) by UGD-glucuronosyltransferase (UGT) 1A1, 1A3, and 2B7 (Sakoda et al., 2025), and the serum concentration of GA30G was much lower than that of GA3S (Takahashi et al., 2019; Sakoda et al., 2024). We investigated the enzymes responsible for the biotransformation to produce GA3S, and identified that SULT2A1 specifically catalyzes the sulfation of the C-3 hydroxyl group of GA (Takahashi et al., 2019). This indicates that in humans, sulfation—rather than glucuronidation—is the primary hepatic detoxification pathway for GA.

### 5.2 Stereoselective metabolism: the resistance of 3-*epi*-GA

The most significant update in our understanding of GA metabolism is the “stereoselectivity” of SULT2A1. As described in the Absorption section, specific intestinal microbiota converts GA into its isomer, 3-*epi*-GA. While these two molecules differ only in the spatial orientation of the hydroxyl group at the C-3 position (GA has a *β* position, while 3-*epi*-GA has an *α*), this slight difference drastically alters their metabolic fate.

We demonstrated that SULT2A1 discriminates between these isomers (Sakoda et al., 2025). While SULT2A1 efficiently converts GA into hydrophilic GA3S, it shows very low activity toward 3-*epi*-GA. Due to steric hindrance at the C-3 position, 3-*epi*-GA is resistant to SULT2A1-mediated sulfation. Consequently, while GA is rapidly metabolized and prepared for excretion, 3-*epi*-GA escapes this hepatic clearance. This “metabolic resistance” allows 3-*epi*-GA to bypass the liver’s detoxification system and persist in the systemic circulation for a prolonged period. This mechanism likely explains why patients who possess 3-*epi*-GA-producing intestinal microbiota (“epimerization phenotype”) are prone to accumulating high levels of active metabolites, even at standard doses of licorice.

## 6 Excretion: biliary regurgitation and renal delivery mechanisms

The excretion process acts as the final gatekeeper in the mechanism of toxicity. Our research suggests that pseudohyperaldosteronism is not merely a side effect of high blood concentrations, but the result of a “transport failure” in the liver (regurgitation) followed by a specific “delivery system” in the kidney. This multi-step mechanism highlights why patients with subtle hepatobiliary dysfunction or those with specific transporter phenotypes are at elevated risk.

### 6.1 Hepatic excretion: MRP2 and the regurgitation mechanism

Under physiological conditions, the liver efficiently clears GL metabolites. After GL is hydrolyzed to GA and absorbed, the liver conjugates GA (mainly to GA3S in humans) to increase water solubility. We previously demonstrated that these conjugates are substrates for multidrug resistance-associated protein 2 (Mrp2/ABCC2), an efflux transporter located on the canalicular membrane of hepatocytes (Makino et al., 2008; Morinaga et al., 2018; Ishiuchi et al., 2019; Ishiuchi et al., 2021). MRP2 pumps these conjugates into the bile, facilitating their excretion into feces. Consequently, in healthy individuals, the systemic blood concentration of these conjugates remains low.

However, this excretion pathway is vulnerable. We found that in rats with drug-induced liver injury or in Eisai hyperbilirubinemic rats (EHBRs) lacking functional Mrp2, the biliary excretion of GL metabolites is severely impaired. Instead of entering the bile, these metabolites accumulate in the hepatocytes and eventually “regurgitate” back into the blood circulation via basolateral transporters (likely Mrp3). This mechanism explains the clinical risk factor we identified: high direct bilirubin (Yoshino et al., 2016; Komatsu et al., 2019). Direct bilirubin is also a substrate for MRP2 (Kamisako et al., 1999). Therefore, an elevated direct bilirubin level serves as a surrogate marker for MRP2 dysfunction or competitive inhibition. In these patients, biliary excretion is compromised, causing GL metabolites to regurgitate into the systemic circulation, thereby flooding the blood and exposing the kidneys to high concentrations of inhibitors.

### 6.2 The “Proximal-Secretion Distal-Absorption” hypothesis

Once these metabolites reach the kidney via blood, a spatial paradox arises. The target enzyme, 11*β*-HSD2, is located in the distal tubules and collecting ducts (Chapman et al., 2013). However, the transporters known to uptake organic anion transporters (OATs) are located primarily in the proximal tubules (Nigam et al., 2015). Based on our series of *in vitro* and animal experiments, we propose the “Proximal-Secretion Distal-Absorption” hypothesis. It is important to note that these renal transport models and luminal entry pathways are not yet fully established in humans and remain hypothetical.

1. Basolateral Entry in Proximal Tubules: We confirmed that while lipophilic GA enters cells *via* passive diffusion, hydrophilic conjugates (such as GA3S, GA30G, and the newly identified 3-*epi*-GA-30-*O*-glucuronide, 3-*epi*-GA30G) cannot easily cross cell membranes. Using HEK293 cells expressing human transporters, we demonstrated that OAT1 (SLC22A6) and OAT3 (SLC22A8) actively uptake these conjugates from the blood into renal cells (Ishiuchi et al., 2019; Sakoda et al., 2025). Since OAT1 and OAT3 are localized to the basolateral membrane of the proximal tubule in humans (Nigam et al., 2015), this is the site of initial entry.
2. Secretion and Transport: After entering the proximal tubular cells, these metabolites are secreted into the tubular lumen (urine) via apical transporters (likely MRP2 or MRP4). They then flow down the nephron in the primary urine.
3. Reuptake in the Distal Nephron: As the primary urine travels to the distal tubules and collecting ducts, water is reabsorbed, concentrating the metabolites. We hypothesize that these concentrated metabolites in the lumen are then reabsorbed into the distal tubular cells (where 11*β*-HSD2 resides) (Chapman et al., 2013). This reuptake is likely mediated by OATPs, such as OATP1A2, which are expressed on the apical membrane of the distal nephron (Roth et al., 2012). Alternatively, as the urine becomes more acidic in the distal segments, the metabolites, along with GA and 3-*epi* GA, may become a non-ionized form and enter the cells via passive diffusion.

This “circular route”—secreted in the proximal tubule to attack the distal tubule from the inside—explains why renal function and urinary concentration mechanisms are critical variables in susceptibility.

### 6.3 Stereoselective excretion of 3-*epi*-GA

The discovery of the stereoisomer 3-*epi*-GA adds another layer of complexity to excretion. As discussed in the Metabolism section, 3-*epi*-GA is resistant to sulfation by SULT2A1 (Sakoda et al., 2025). This resistance prevents it from being efficiently cleared into the bile, extending its half-life in the blood. Furthermore, our latest data suggest that while 3-*epi*-GA itself is not a substrate for OATs, its glucuronide conjugate, 3-*epi*-GA30G, is recognized and transported by OAT3. This implies that even if the liver fails to clear 3-*epi*-GA, it can eventually be conjugated to 3-*epi*-GA30G, transported into the kidney, and inhibit 11*β*-HSD2. The persistence of 3-*epi*-GA in the body acts as a “slow-release reservoir,” providing a steady source of toxic metabolites to the kidney long after the drug was ingested. Importantly, GA30G and 3-*epi*-GA30G are the only metabolites consistently detected in human urine (Sakoda et al., 2024). This suggests that urinary screening could serve as a non-invasive biomarker to estimate the individual metabolic phenotype of 3-*epi*-GA without the need for blood sampling. However, further investigation into the precise kinetic affinities of these conjugates for renal transporters is necessary to fully validate this diagnostic potential.

## 7 Limitations

This review has several limitations. First, many of the proposed mechanisms, including renal transport models, rely heavily on preclinical data (*in vitro* assays and animal models). Second, clinical findings regarding 3-*epi*-GA are currently based on limited observational cohorts utilizing single-point sampling, and sequential time-course data are lacking. Finally, the findings primarily derive from patients using Japanese Kampo medicine, requiring external validation in populations exposed to licorice through diet or other traditional medicines.

## 8 Conclusion: toward precision medicine for licorice-induced pseudohyperaldosteronism

The understanding of licorice-induced pseudohyperaldosteronism has evolved significantly from a simple dose-dependent model to a complex interaction involving pharmaceutical properties, intestinal microbiota, and host physiology. While the daily dosage of licorice remains a baseline risk factor, recent evidence explains the clinical paradox of why severe side effects occur in some patients taking low-dose formulations, while others tolerate high-dose formulations (Table 1).

**TABLE 1:** Clinical risk and protective factors for licorice-induced pseudohyperaldosteronism.

Category	Factor	Pharmacokinetic/Clinical rationale
Risk factors	High daily dosage	Dose-dependent inhibition of renal 11*β*-HSD2
	Long-term use (> 30 days)	Accumulation of licorice metabolites in the body
	Older age	Age-related decline in 11*β*-HSD2 activity and decreased physiological reserve
	Constipation	Prolonged colonic transit time increases the absorption of microbiota-generated metabolites
	Concomitant potassium-wasting diuretics or glucocorticoids	Synergistic potassium excretion (*e.g.*, loop or thiazide diuretics); systemic glucocorticoids aggravate the inhibition of 11*β*-HSD2
	Hypoalbuminemia	Increases the free fraction of glycyrrhetinic acid and 3-*epi*-GA in systemic circulation
	Epimerization phenotype	Specific intestinal microbiota convert glycyrrhizin to 3-*epi*-GA, which evades hepatic sulfation
Protective factors	Formulation matrix effects	Low pH (*e.g.*, Schisandra fruit) limits glycyrrhizin solubility and extraction efficiency
	Enzymatic inhibition by baicalin	Baicalin (in Scutellaria root) inhibits bacterial *β*-glucuronidase, reducing the conversion of glycyrrhizin to absorbable GA
	Concomitant potassium-sparing medications	Aldosterone blockers, angiotensin-converting enzyme inhibitors, or angiotensin II receptor blockers counter potassium loss

11*β*-HSD2: 11*β*-hydroxysteroid dehydrogenase type 2, GA: 18*β*-glycyrrhetinic acid.

Pharmaceutical Factors: Formulation Matters We now recognize that the “prescribed dose” does not equal the “absorbed dose.” Two pharmaceutical mechanisms regulate this bioavailability: pH-dependent solubility and enzymatic competition. In shoseiryuto formulation, organic acids from SF lower the pH, precipitating GL and reducing its concentration in the decoction. In formulations such as saireito and bofutsushosan, baicalin from SR competes for bacterial *β*-glucuronidase that limits the release of the active aglycone, GA. Conversely, formulations lacking these inhibitory factors, such as shakuyakukanzoto and yokukansan, allow for efficient hydrolysis and absorption, presenting a higher risk profile relative to their licorice content.

Host Factors: The “Epimerization” Phenotype The most critical update since 2021 is the identification of 3-*epi*-GA and the defining role of the intestinal microbiota. Specific intestinal microbiota (e.g., *C*. *innocuum*) do not merely hydrolyze GL but actively convert it into this stereoisomer. Unlike typical GA, 3-*epi*-GA is resistant to hepatic sulfation by SULT2A1, leading to prolonged retention in the body. This “epimerization phenotype” likely defines the high-risk patient group that was previously difficult to identify based on dosage alone.

Integration with Age and Physiology Furthermore, the risk is amplified by host physiological states. Hypoalbuminemia, common in the elderly, increases the fraction of free GA and 3-*epi*-GA. This allows the compounds to bypass restricted distribution and potentially enter the renal tubules via glomerular filtration—a mechanism bypassing normal distribution. When combined with the age-related physiological decline of the protective enzyme 11*β*-HSD2, these factors explain the steep increase in pseudohyperaldosteronism incidence among older adults.

Clinical Implications Consequently, clinical risk assessment must move beyond simply counting grams of licorice. We propose a precision medicine approach that considers:

1. The Formulation: Check for the presence of SR (risk reduction) or SF (risk reduction).
2. The Patient: Evaluate age, albumin levels, and bowel habits (constipation increases absorption).
3. The Biomarkers: In the near future, monitoring urinary 3-*epi*-GA30G could serve as a definitive method to identify patients with high metabolic susceptibility before severe hypokalemia occurs.

By integrating these pharmaceutical and metabolic insights, clinicians can maximize the therapeutic benefits of herbal medicine containing licorice while effectively minimizing the risk of this preventable adverse effect. Furthermore, because licorice is widely available as not only over-the-counter products but foods, public awareness and direct consumer guidance are necessary to prevent inadvertent overconsumption, especially for individuals with existing risk factors.

## 9 List of the compounds studied

3-*epi*-18*β*-glycyrrhetinic acid, 3-*epi*-18*β*-glycyrrhetinic acid-30-*O*-glucuronide, galacturonic-glycyrrhizin, 18*β*-glycyrrhetinic acid, 18*β*-glycyrrhetinic acid-3-*O*-glucuronide, 18*β*-glycyrrhetinic acid 3-*O*-sulfate, 18*β*-glycyrrhetinic acid 30-*O*-glucuronide.
